# Advances in the application of nanoparticles for the diagnosis and treatment of diabetic cardiomyopathy

**DOI:** 10.3389/fcell.2026.1912847

**Published:** 2026-08-21

**Authors:** Jia Feng, Yuqing Huang, Yufeng He, Chenhao Deng, Zongzhe Jiang, Qifu Li, Yu Li, Xiaorong Lan, Yong Xu

**Affiliations:** 1 Department of Endocrinology and Metabolism, The Affiliated Hospital of Southwest Medical University, Luzhou, China; 2 Metabolic Vascular Diseases Key Laboratory of Sichuan Province, Southwest Medical University, Luzhou, China; 3 Sichuan Clinical Research Center for Nephropathy, Luzhou, China; 4 Sichuan-Chongqing Joint Key Laboratory of Metabolic Vascular Diseases, Luzhou, China; 5 Department of Cardiology, The Affiliated Hospital of Southwest Medical University, Luzhou, China; 6 Department of Endocrinology, The First Affiliated Hospital of Chongqing Medical University, Chongqing, China; 7 CAS Key Laboratory of Nutrition and Metabolism, Shanghai Institute of Nutrition and Health, University of Chinese Academy of Sciences, Chinese Academy of Sciences, Shanghai, China; 8 Institute of Stomatology, Southwest Medical University, Luzhou, China; 9 Luzhou Key Laboratory of Oral and Maxillofacial Reconstruction and Regeneration, The Affiliated Stomatological Hospital, Southwest Medical University, Luzhou, China

**Keywords:** diabetic cardiomyopathy, nanoparticles, responsive release, targeted delivery, theranostics

## Abstract

As a frequent and serious cardiovascular complication of diabetes mellitus, diabetic cardiomyopathy (DCM) typically presents with an asymptomatic onset during its initial phase, followed by gradual worsening over time. Currently, no universally accepted and effective DCM-specific targeted therapeutic strategy is available. Recently, nanotechnology has gained recognition as a viable strategy to address the shortcomings of traditional treatments, leveraging its size-dependent effects, large specific surface area, and ability to integrate multiple functions. This article presents a structured summary of recent developments regarding nanoparticle utilization for diagnostic and therapeutic purposes in DCM. First, from a functional perspective, we analyze the core advantages of nanoparticles in improving pharmacokinetic profiles, enhancing drug stability and bioavailability, facilitating targeted myocardial delivery, enabling pathological microenvironment-responsive release, and constructing theranostic platforms. Second, according to material type, we discuss the current research status and the operational mechanisms of different nanocarrier systems—including polymeric nanoparticles, liposomes, inorganic nanoparticles, and extracellular vesicles—in antifibrotic therapy, antioxidation intervention, regulation of energy metabolism, and early diagnostic assessment. Finally, based on current limitations and successful experience from other cardiovascular diseases we outline future directions, including biomimetic design, upgraded targeting strategies, diversified administration routes, safety evaluation, and combined applications with hydrogels or myocardial patches. Although nanotechnology has shown promising potential in animal models of DCM, clinical translation still requires continued breakthroughs in material innovation, quality control, and long-term safety validation.

## Introduction

Globally, 589 million adults aged 20–79 years currently live with diabetes, and this number is projected to reach 853 million by 2050 (International Diabetes Federation, 2025). In China, an estimated more than 233 million people have diabetes (Zhou et al., 2025). Among individuals with type 2 diabetes, cardiovascular disease constitutes the leading cause of both morbidity and mortality (Solini, 2025). Diabetic cardiomyopathy (DCM) is defined as myocardial structural or functional abnormalities occurring in the setting of type 2 diabetes without concurrent coronary artery disease, hypertension, or obesity (Radzioch et al., 2024). The early stages of DCM often present with occult diastolic dysfunction, including reduced myocardial compliance and increased left ventricular stiffness. As the disease progresses, myocardial cell injury and interstitial fibrosis further worsen, ultimately leading to systolic dysfunction and heart failure (Galeone et al., 2024; Chen et al., 2025). In recent years, clinical imaging and molecular biology studies have increasingly revealed that the pathological mechanisms of DCM are highly complex, involving a coordinated dysregulation of multiple metabolic and stress pathways rather than a single causative factor (Chen et al., 2025).

At the metabolic level, hyperglycemia, hyperlipidemia, and insulin resistance shift myocardial energy substrate selection from a relatively flexible state to one dominated by fatty acid oxidation, resulting in decreased energy utilization efficiency and an increased lipotoxic burden (Jia and Shi, 2026; Satyanarayana et al., 2026). Meanwhile, prolonged myocardial exposure to metabolic stress induces mitochondrial dysfunction, which diminishes ATP synthesis and aggravates cellular stress (Pham et al., 2025; Dhalla et al., 2014). On this basis, oxidative stress and inflammatory signaling are continuously activated, and excessive reactive oxygen species (ROS) can inflict damage on cellular structures and DNA, thereby exacerbating inflammatory responses through pathways including NF-κB and NLRP3, forming a vicious cycle (Yaghooti et al., 2025; Saleh et al., 2025). Furthermore, dysregulated intracellular and extracellular signaling mediated by advanced glycation end products and their receptor serves as a critical pathological driver of myocardial stiffening, fibrotic changes, and inflammatory responses (Kitazawa and Kitazawa, 2026; Gupta et al., 2025). The diabetic myocardium also exhibits varying degrees of apoptosis, altered autophagy, and disruption of organelle homeostasis, which collectively contribute to myocardial remodeling, functional decline, and disease progression (Luo et al., 2025).

Despite the growing understanding of DCM pathophysiology, clinical translation remains challenging. Current treatment strategies rely mainly on glycemic control and conventional heart failure medications, but such interventions are often initiated late and cannot reverse established myocardial fibrosis or mitochondrial dysfunction (Chen et al., 2025; Khattab et al., 2025). Moreover, DCM has an insidious onset, lacking early specific symptoms and sensitive biomarkers, leading to a delayed diagnostic window. Most patients are already at an irreversible stage of ventricular remodeling by the time of diagnosis (Deng et al., 2023; Balakrishnan et al., 2025). Therefore, developing novel theranostic approaches that enable both early diagnosis and targeted intervention is an urgent need in DCM research.

Leveraging its unique size effects, high specific surface area, and multifunctional integration capabilities, nanotechnology presents a new platform for the precise diagnosis and therapy of DCM (Luo et al., 2026; Saadh et al., 2025a). Compared with conventional drugs, nanoparticles can remarkably optimize pharmacokinetic profiles, extend blood circulation time, and accumulate in infarcted myocardial tissue via active or passive targeting strategies (Saadh et al., 2025a). More importantly, stimuli-responsive nanoplatforms enable the on-demand release of therapeutic agents upon sensing pathological microenvironmental cues (e.g., high ROS, acidic pH), thereby improving the therapeutic index while lowering systemic toxicity (Xue et al., 2026). Furthermore, some inorganic nanomaterials possess intrinsic catalytic antioxidant or imaging-enhancing properties, enabling direct participation in oxidative stress regulation or non-invasive visualization.

**FIGURE 1:**
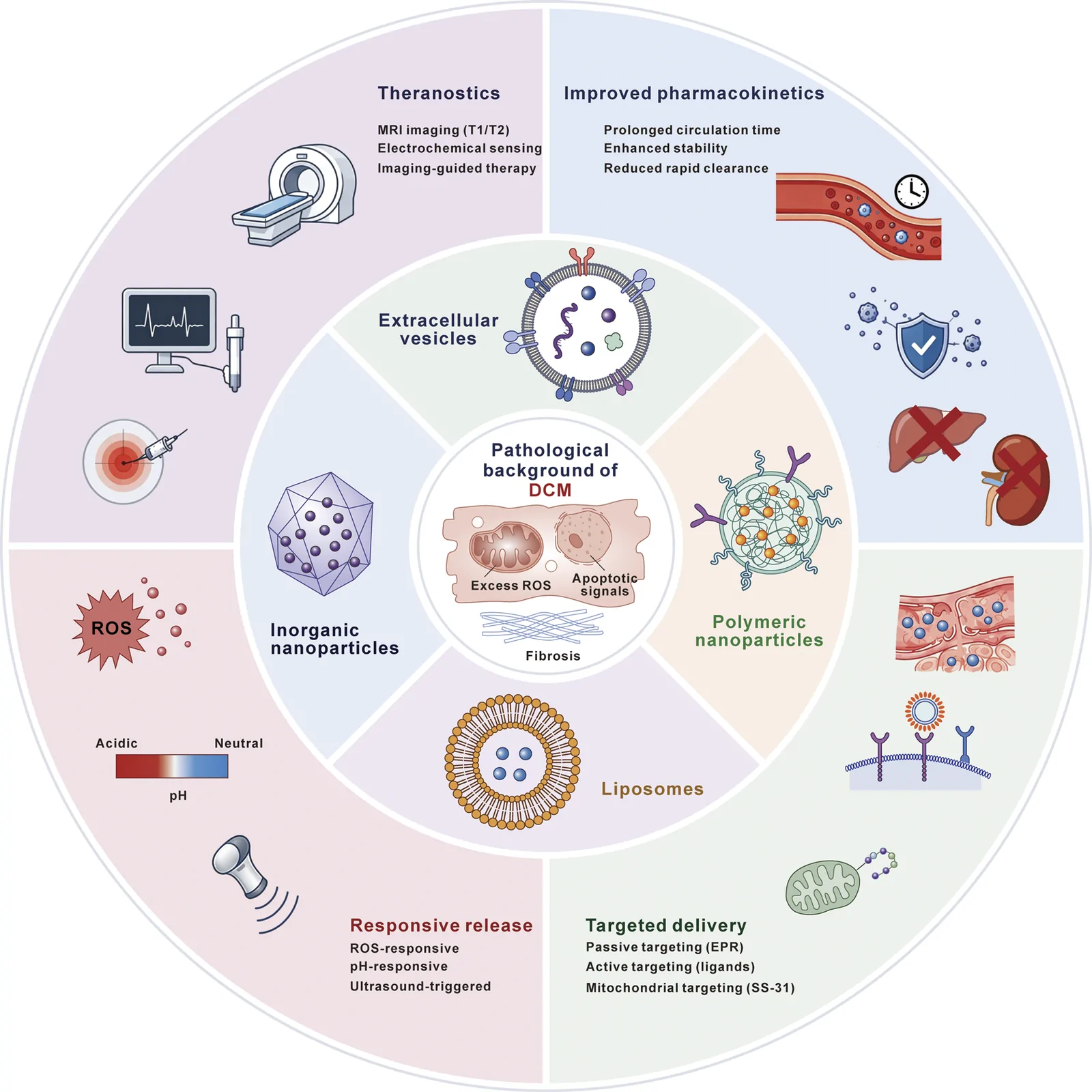
Functional integration and material strategies of nanoparticles in DCM diagnosis and therapy. The inner layer shows the main pathological features of the DCM myocardium (mitochondrial dysfunction, oxidative stress, fibrosis); the middle layer presents schematic structures of four typical nanomaterials; the outer layer indicates four core functions. Dashed arrows indicate correspondences between material types and functional modules (a given material may have multiple functions). Abbreviations: MRI, magnetic resonance imaging. DCM, diabetic cardiomyopathy; ROS, reactive oxygen species; EPR, enhanced permeability and retention effect.

In this context, the present review offers a systematic synthesis of recent progress in nanotechnology for DCM across two dimensions: functional characteristics (improving pharmacokinetics, targeted delivery, responsive release, theranostics) and material types (polymeric nanoparticles, liposomes, inorganic nanoparticles, extracellular vesicles), and compares the advantages and disadvantages of different nanoplatforms (Figure 1). Finally, drawing on applications in other cardiovascular diseases, we discuss the prospects of biomimetic nanoparticles, local delivery systems, and intelligent theranostic platforms in DCM, with the goal of guiding future research.

## Characteristics of nanoparticles in DCM theranostics

**FIGURE 2:**
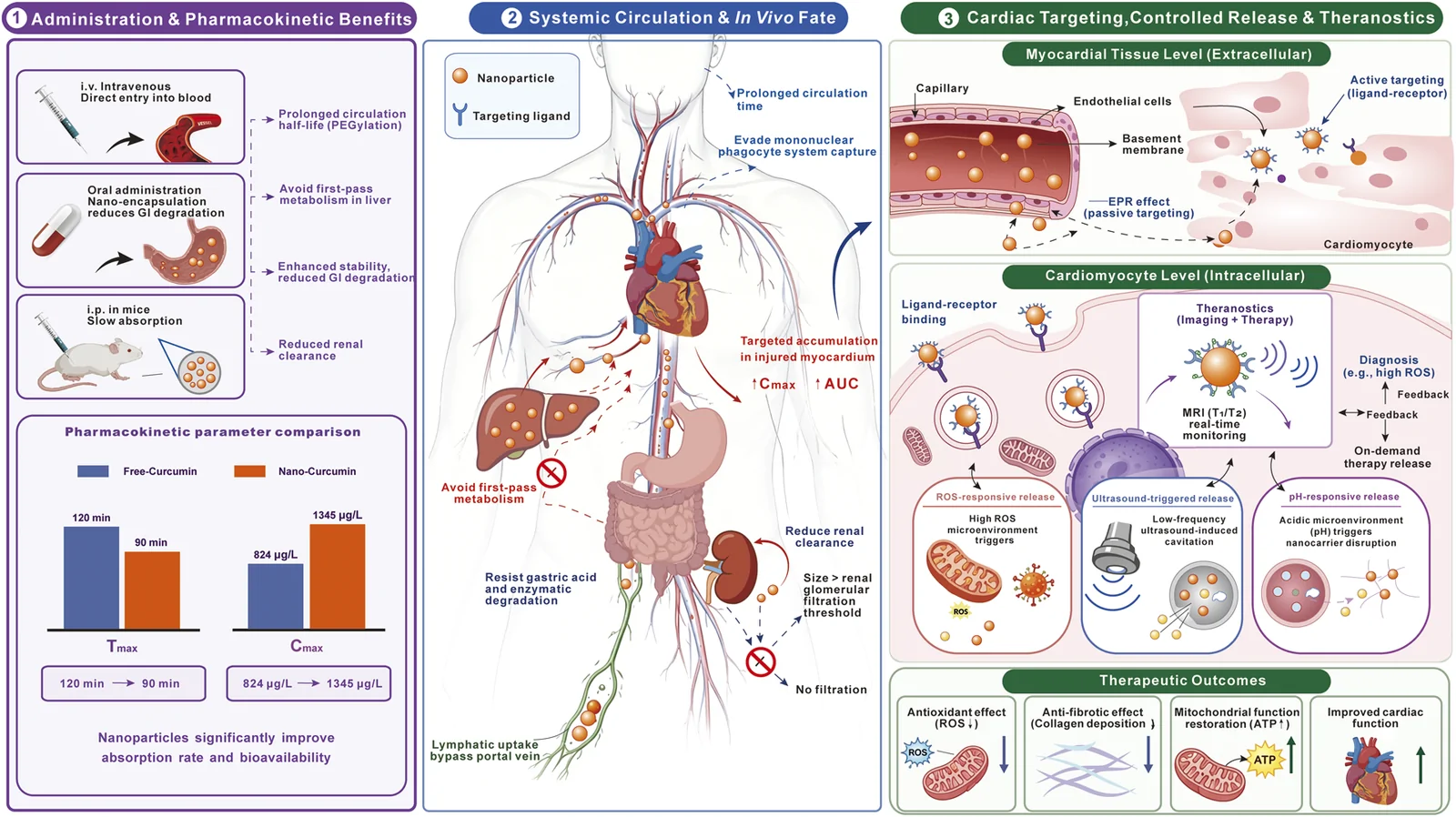
*In vivo* delivery pathways, myocardial targeting, and drug release strategies of nanoparticles in DCM. The left panel illustrates three administration routes (intravenous injection, oral administration, and intraperitoneal injection). Taking curcumin nanoparticles as an example (data from Abdel-Mageid et al. (2018)), the nanoformulation achieves a shorter time to peak concentration and a higher maximum concentration compared with the free drug, indicating that nanoencapsulation improves both the absorption rate and bioavailability. The main mechanisms by which nanoparticles improve pharmacokinetic profiles—including prolonged circulation time, reduced gastrointestinal degradation, avoidance of first-pass hepatic metabolism, and diminished renal clearance—are also summarized on the left. In the central human silhouette, nanoparticles (orange-red spheres) exhibit extended circulation within the vasculature; some evade rapid clearance by the liver and kidneys owing to their favorable size and surface modifications, while orally administered particles remain stable in the gastrointestinal tract. The majority of nanoparticles are targeted to and accumulate in the damaged myocardium. The magnified panel on the right depicts nanoparticles entering the myocardium through passive targeting (EPR effect) and active targeting (via surface ligands), followed by drug release triggered by ROS or ultrasound stimulation. Certain platforms additionally integrate MRI imaging with therapeutic functions, enabling theranostic applications. Specific materials and the corresponding mechanisms are discussed in Sections 2, 4. Abbreviations: T_max_, time to maximum concentration; _Cmax_, maximum plasma concentration; AUC, area under the concentration–time curve; EPR, enhanced permeability and retention effect; MRI, magnetic resonance imaging; ROS, reactive oxygen species.

As innovative drug delivery platforms, nanoparticles exhibit unique advantages in the treatment of DCM. Their core characteristics include: improving pharmacokinetics by effectively prolonging drug circulation time and enhancing stability through encapsulation; achieving targeted delivery by surface modification to actively accumulate in diseased cardiac tissue, thereby enhancing therapeutic efficacy and minimizing systemic toxicity, while enabling stimulus-triggered drug liberation in response to alterations in the myocardial microenvironment (e.g., pH, ROS), thereby maintaining long-term effective concentrations; and facilitating theranostic applications, since certain nanomaterials are capable of co-delivering imaging contrast agents and therapeutic drugs, thereby enabling real-time monitoring and treatment assessment. These features collectively overcome the multiple bottlenecks of conventional drug delivery in treating this complex disease, providing a new strategy for precise and efficient intervention in DCM. These nanoparticles enter the body via different administration routes, prolong circulation time, target the damaged myocardium, and achieve responsive release and theranostics (Figure 2).

### Improved pharmacokinetics

Serving as drug delivery platforms, nanoparticles can optimize the pharmacokinetic properties of conventional therapeutic agents through diverse mechanisms, thereby substantially enhancing their therapeutic efficacy and safety profiles. To begin with, encapsulating drugs into nanocarriers can substantially extend their residence time within systemic circulation. For example, He et al. loaded liraglutide into tannic acid–aluminium ion (TA/Al^3+^) nanoparticles. The sustained-release properties of the nanoparticles significantly extended the *in vivo* circulation time of the drug. Unconjugated liraglutide exhibits a half-life of merely about 13 h, necessitating daily administration. In contrast, a single intraperitoneal injection of this nanoformulation (NP-3) maintained effective plasma concentrations for up to 6 days, and the fluorescence intensity in blood was 30 times that of the free drug at 144 h, greatly improving bioavailability and hypoglycemic efficacy (He et al., 2020).

Secondly, nanoparticles can substantially improve the dissolution behavior and stability of drugs with poor aqueous solubility, thus facilitating faster absorption and elevating peak plasma concentrations. In the study by Abdel-Mageid et al., compared with ordinary curcumin, nano-curcumin suspension in rats reached peak concentration sooner (90 min vs. 120 min) and with a significantly higher maximum plasma concentration, indicating greatly improved absorption efficiency and systemic exposure (Abdel-Mageid et al., 2018).

Furthermore, nanocarriers (e.g., liposomes) can alter drug biodistribution and avoid premature metabolism. Although resveratrol has multiple biological activities, its oral bioavailability is very low owing to poor water solubility (<1 μM) and rapid *in vivo* metabolism (half-life 8–14 min) (Amri et al., 2012; Yücel et al., 2018; Bonechi et al., 2012). After encapsulating resveratrol in liposomes, Alanazi et al. not only improved its stability in aqueous solution but also provided protection through the lipid bilayer, reducing gastrointestinal degradation and first-pass hepatic effects, allowing sufficient drug to reach target tissues (Alanazi et al., 2024).

Despite the notable pharmacokinetic advantages conferred by the aforementioned nanoformulations, a significant translational gap remains in the design of dosing regimens in current DCM nanomedicine studies, the vast majority of *in vivo* experiments employ single or weekly multiple intravenous/intraperitoneal injection schedules, yet DCM is a chronic disease requiring lifelong management, and frequent injection-based administration faces practical clinical obstacles, including poor patient compliance, the burden of repeated hospital visits, and an increased risk of infection. Nanotechnology offers multiple possibilities for optimizing administration routes: oral administration is the most convenient, but is only applicable to nanocarriers that remain stable in the gastrointestinal environment; subcutaneous implantable sustained-release formulations can achieve ultra-long-term release over weeks to months, but require surgical implantation and removal; transdermal patches combine the advantages of non-invasiveness and sustained delivery, yet are limited by the skin barrier and drug-loading capacity. These routes differ in their respective trade-offs among convenience, compliance, and sustainability, with the optimal choice also varying according to the nanocarrier type. More importantly, most current studies only report pharmacokinetic parameters from single-dose administration, lacking systematic evaluation of nanoparticle accumulation, metabolic clearance, and cumulative toxicity after long-term repeated dosing, the absence of multi-dose pharmacokinetic data not only limits the assessment of long-term safety but also hinders the clinical translation of dosing regimens. Future studies should conduct systematic multi-dose pharmacokinetic-pharmacodynamic studies in DCM animal models to provide an experimental basis for the design of clinical dosing regimens.

### Targeted delivery

The targeted delivery capability of nanoparticles is a key factor in achieving precise and safe treatment of DCM. Based on the targeting mechanism, it can be divided into passive and active targeting, each with distinct design concepts and application advantages in the complex pathological environment of DCM.

Passive targeting relies mainly on the enhanced permeability and retention (EPR) effect, whereby nanoparticles within a specific size range can pass through the loose vascular endothelial gaps of diseased tissue and become retained in the interstitium (Alghamdi et al., 2022). However, the validity of the EPR effect as a targeting mechanism in DCM is subject to considerable caveats. Distinct from tumor-characteristic structurally aberrant neovessels and markedly widened endothelial gaps, DCM-associated myocardial injury presents a diffuse pattern. Its microvascular lesions mainly manifest as basement-membrane thickening, endothelial dysfunction and microvascular rarefaction instead of structural vascular leakage (Knapp et al., 2019; Shi and Vanhoutte, 2017). Under diabetic conditions, persistent hyperglycemia triggers oxidative stress and inflammatory cascades, which further downregulate and disrupt endothelial junction proteins (such as VE-cadherin and claudin-5), ultimately causing excessive elevation of endothelial barrier permeability (Zhang et al., 2023). These pathological changes exert two-sided effects on nanoparticle extravasation. The enhanced permeability in theory promotes passive diffusion of nanoparticles across endothelial gaps into the myocardial interstitium. Nevertheless, DCM concurrently induces microvascular rarefaction, basement-membrane thickening and perivascular fibrosis; these structural abnormalities hinder deep-tissue penetration of nanoparticles (Farhangkhoee et al., 2006). More importantly, injured endothelium displays dysregulated expression of adhesion molecules. Although this phenomenon boosts the non-specific adsorption of nanoparticles, it paradoxically initiates endothelial phagocytosis. As a result, part of nanoparticles is retained within endothelial layers and fail to access cardiomyocytes (Papademetriou et al., 2014). Additionally, upregulated ROS and pro-inflammatory cytokines in DCM drive endothelial-to-mesenchymal transition, which remodels microvascular barrier integrity and transport capacity (Kang et al., 2025). Accordingly, only marginal permeability differences exist between healthy and diseased myocardial tissues. In this context, passive-targeting strategies alone fail to build up sufficient concentration gradients of therapeutic nanoparticles within damaged myocardium of DCM patients. Besides, systemic endothelial dysfunction commonly occurs among DCM patients, rendering the enhanced permeability and EPR highly unpredictable. Taken together, passive targeting yields unsatisfactory therapeutic outcomes for DCM, and it is frequently integrated with active-targeting approaches to improve delivery efficiency.

To overcome the above-mentioned barrier challenges, active targeting strategies can be implemented through multiple approaches. First, nanoparticles can be functionalized with ligands targeting receptors overexpressed on endothelial cells, enabling their initial anchorage to the diseased microvascular endothelium followed by receptor-mediated transcytosis across the endothelial layer. This approach effectively bypasses the disrupted intercellular junction pathways, shifting the delivery paradigm from passive diffusion through damaged barriers to active exploitation of endothelial transport machinery, thereby improving transendothelial delivery efficiency (Liu et al., 2026). Second, certain nanoplatforms are capable of not only delivering therapeutic payloads but also directly ameliorating microvascular injury—exemplified by PSS-NP, which restores coronary microvascular architecture and function through the enhancement of endothelial function, suppression of inflammation, and alleviation of oxidative stress (Mao et al., 2020). Third, biomimetic membrane coating strategies have emerged as a promising approach to surmount these barriers. By preserving the intrinsic inflammatory tropism receptors of the source cell membranes, these biomimetic nanoparticles are able to actively recognize and traverse activated diseased endothelium, while simultaneously shielding themselves from phagocytic clearance by the reticuloendothelial system (Gao et al., 2020). Although direct applications of this strategy in DCM remain limited, macrophage membrane-coated nanoparticles have been investigated in other cardiovascular diseases. Leveraging their superior immune evasion capacity and inherent inflammatory tropism, these biomimetic nanoplatforms significantly enhance drug accumulation in diseased tissues, positioning them as a promising direction for the treatment of cardiovascular complications (Li et al., 2026).

After successfully crossing the microvascular barrier, nanoparticles require further precise delivery to target cardiac cells and even specific subcellular organelles. Surface functionalization of nanoparticles with specific ligands facilitates the specific recognition and binding of overexpressed receptors and biomarkers on DCM-injured myocardium and specific organelles, ultimately realizing accurate targeted delivery (Xu et al., 2025a). At the cardiomyocyte targeting level, Liu et al. constructed dual-functionalized superparamagnetic iron oxide (SPIO) nanoparticles modified with cardiac homing peptide (CHP) and matrix metalloproteinase 2 (MMP2) antibody (Liu et al., 2024). This dual modification strategy enables the early and sensitive diagnosis of DCM-induced myocardial fibrosis. At the subcellular level, Du et al. engineered ultra-small iron oxide (ESIO) nanoparticles conjugated with CHP and the mitochondrial-targeting peptide SS-31 (Du et al., 2025). The fabricated nanocarriers can precisely deliver Argonaute-2 (AGO-2) protein into dysfunctional cardiomyocyte mitochondria, thereby directly reversing mitochondrial oxidative stress and ameliorating myocardial injury. Although the aforementioned targeting strategies can deliver nanoparticles to diseased cardiomyocytes in DCM, such approaches must circumvent nanoparticle phagocytosis by macrophages. In this context, macrophage-directed therapeutic targeting represents a more accessible and selective strategy (Weissleder et al., 2014; Lameijer et al., 2013). For example, Jia et al. modified gold nanoparticles (AuNPs) with an antago-miR155 to confer macrophage-targeting capability (Jia et al., 2017). The relatively small size of the AuNPs further facilitates their phagocytic uptake by macrophages. These nanoparticles were shown to accumulate in both resident and recruited macrophages in the diseased heart, thereby attenuating cardiac inflammation, apoptosis, and fibrosis. Compared with non-targeted nanoparticles, this active targeting approach markedly diminishes non-specific sequestration by the reticuloendothelial system (e.g., liver and spleen), reduces systemic toxicity, and significantly enhances both the local effective drug concentration and the therapeutic index.

These functionalized nanoparticles enable targeted drug release in the myocardium or specific cells, thereby reducing systemic toxicity. Moreover, as a platform, nanoparticles can carry multiple drugs simultaneously, enabling multimodal synergistic therapy and providing new tools to address the pathological mechanisms of DCM. However, even if nanoparticles successfully cross the microvascular barrier and are taken up by myocardial cells, their precise delivery to the cytoplasm or specific subcellular organelles still faces the key bottleneck of endosome/lysosome escape (Chahal et al., 2026). After entering the cell, nanoparticles are mainly taken up through endocytosis, with the vast majority remaining in endosome/lysosome compartments. They undergo degradation or inactivation under acidic conditions (pH 4.5–5.5) and the action of various hydrolytic enzymes, resulting in extremely low efficiency for cytoplasmic release or mitochondrial delivery. Studies have shown that endosome escape efficiency is usually less than 5%, with some reports even only 1%–2% (Mrksich et al., 2024; Conti et al., 2026). There are various mature nanotechnology techniques available for reference in addressing the bottleneck of endosome escape, including introducing membrane fusion lipids to promote membrane destabilization, modifying pH sensitive transmembrane peptides to induce membrane lysis in acidic environments, and constructing proton sponge effect polymers to expand the endosome through osmotic pressure (Ahmad et al., 2019). However, the systematic application of the above strategies in DCM treatment is still blank. In future research, when designing subcellular targeted nanoparticles, the efficiency of endosome escape should be considered as one of the key evaluation indicators, and the integration of endosome escape module and mitochondrial targeting module on the same nanoplatform should be explored to truly achieve precise delivery from blood vessels, cells, and subcellular.

### Responsive release

Responsive release, also known as intelligent or controlled release, refers to the design of nanostructures that enable loaded drugs or imaging agents to be released at a predetermined rate, at a specific site, or in response to internal or external stimuli. The core is on-demand release, relying on two main mechanisms: endogenous and exogenous stimulus-responsive release (Du et al., 2025; Xu et al., 2025b; Ren et al., 2026).

The myocardial tissue in DCM presents a pathological microenvironment characterized by acidic pH, high ROS levels, and overexpression of matrix metalloproteinases, providing conditions for endogenous triggering. Xue et al. constructed tellurium-bridged covalent organic frameworks, in which dynamic tellurium–tellurium bonds are specifically oxidatively cleaved in a high-ROS microenvironment. This process not only converts ROS into harmless by-products but also triggers framework dissociation, exposing hydrazone groups for *in situ* capture of glycated hemoglobin. When ROS levels recover, the tellurium bonds can be reversibly reduced to self-repair, endowing the system with dynamic regulatory features of on-demand activation and feedback inhibition (Xue et al., 2026). Nie et al. achieved a paradigm shift in signal release using fluorinated carbon nanoprobes: ROS response does not trigger drug diffusion but rather alters the probe’s surface hydrophilicity/hydrophobicity balance and charge, driving selective myocardial enrichment, reducing transverse relaxation time from approximately 28 ms–22 ms, and recovering >85% of the signal after glutathione reduction, offering a new mechanism for recyclable signal regulation (Nie et al., 2023).

Exogenous stimuli-responsive release has also been used for spatiotemporally precise control, with low-frequency ultrasound (LFUS) attracting great attention due to its non-invasiveness and deep penetration. Gao et al. constructed C_3_F_8_-filled PLGA nanobubbles loaded with fibroblast growth factor 21 (FGF21). Under ultrasound irradiation, the cavitation effect triggered instantaneous rupture and drug release, and the sonoporation effect transiently increased microvascular and cell membrane permeability. In model mice, myocardial drug levels increased approximately threefold relative to those in the group not exposed to ultrasound, accompanied by marked downregulation of atrial natriuretic peptide, connective tissue growth factor, and caspase-3 mRNA expression (Gao et al., 2021). This strategy uses physical signals instead of endogenous chemical triggers, avoiding interference from individual microenvironmental heterogeneity in release consistency.

In summary, endogenous and exogenous response strategies have demonstrated advantages in on-demand drug delivery, signal control, and spatiotemporally precise intervention. Endogenous systems autonomously sense and respond to microenvironmental changes with feedback regulation, while exogenous responses offer programmable remote drug release, both enhancing myocardial utilization and reducing systemic toxicity. Current challenges include difficulties in matching endogenous response thresholds to individual dynamic fluctuations, which often result in release kinetics suboptimally aligned with disease stages. In addition, exogenous stimulation is limited by insufficient penetration depth and imprecise focusing accuracy within the beating heart, accompanied by attendant risks of off-target activation. Furthermore, the long-term metabolic fate of nanocarriers and the safety profile of repeated administrations remain to be systematically investigated. Future efforts should develop multi-logic-gated carriers coupling pH, ROS, blood glucose, and other multidimensional signals to improve targeting accuracy; introduce closed-loop feedback systems integrating wearable monitoring with nanoplatforms for dynamic adjustment; and explore combined endogenous–exogenous strategies to construct an endogenous-sensing/exogenous-amplification regulatory network, advancing therapy towards personalized, intelligent, and full-disease-course control.

### Theranostics

Theranostics represents a key direction in nanomedicine, combining both diagnosis and therapy within one nanoplatform, thereby facilitating early disease identification, targeted intervention, and real-time assessment of treatment response (Zheng et al., 2025). DCM has an insidious onset with unclear early symptoms, easily progressing to severe complications such as heart failure. By designing intelligent nanosystems that respond to the disease microenvironment or external field regulation, theranostic strategies are expected to simultaneously achieve lesion visualization, targeted drug delivery, and treatment feedback, providing a new path for precise DCM management (Deng et al., 2023). Current research focuses mainly on two directions: *in vitro* biosensing and *in vivo* imaging diagnosis.

*In vitro* biosensing centres on the highly sensitive detection of DCM-related biomarkers in body fluids such as blood. Kumar et al. developed a water-soluble electrochemiluminescence (ECL) biosensor based on carboxylated graphitic carbon nitride (g-C_3_N_4_) nanoparticles (CNNPs) (Kumar et al., 2025). Following electrophoretic deposition modification, the ECL signal was enhanced by approximately 2.4-fold. The resulting detection limits were determined to be 0.91 μM for H_2_O_2_, 642 μM for glucose, and 135 μM for lactate. By continuously monitoring H9c2 cardiomyocytes under high-glucose stress for 72 h, the metabolic trajectory revealed an inverse relationship between glucose consumption and lactate accumulation, along with dynamic changes in oxidative stress levels. The sensor has the advantages of integration and portability, suitable for point-of-care rapid screening. However, the study was validated only with cell cultures, not with serum or plasma samples, nor was it compared with traditional biochemical methods or commercial sensors, so its clinical reliability requires further validation. Current nanoparticle-based biosensing research for cardiovascular disease mostly focuses on myocardial infarction, with relatively few studies on DCM; its applicability and accuracy need more experimental confirmation (Saadh et al., 2025b).

*In vivo* imaging diagnosis refers to visualizing early pathological changes (e.g., fibrosis, mitochondrial damage) in living organisms. The primary nanoparticle-based imaging modalities currently available are magnetic resonance imaging (MRI) and ultrasound imaging. Among these, MRI serves as the gold standard for evaluating left ventricular volume, function, and mass. This approach enables comprehensive assessment of cardiac chamber dimensions, left ventricular ejection fraction (LVEF), myocardial mass distribution patterns, and tissue properties (Mordi, 2019). However, traditional heavy metal (gadolinium, iron)-containing contrast agents have safety risks such as nephrotoxicity and cannot monitor ROS at the molecular level, only yielding positive results when perfusion defects appear in the late stages of DCM (Idée et al., 2014; Wahsner et al., 2019; Villaraza et al., 2010). Nie et al. developed a metal-free, ROS-responsive T_2_-weighted MRI nanoprobe (RCMN) based on fluorinated carbon nanosheets (FCNs) covalently grafted with TEMPO groups and multiple hydroxyl groups (Nie et al., 2023). In a DCM mouse model, RCMN briefly resided in ROS-rich areas of the myocardium, causing MRI signal changes and advancing the diagnostic window by 8 weeks compared with traditional gadolinium agents (from 16 weeks to 8 weeks). Additionally, the TEMPO groups effectively scavenged some excess ROS, achieving theranostics. Limitations of this study include weak ^19^F signals due to equipment sensitivity and material solubility, preventing ideal ^19^F-MRI imaging. Furthermore, long-term *in vivo* metabolic behavior and safety of multi-cycle administration need systematic evaluation. Future efforts should optimize fluorination degree and nanosheet structure to enhance ^19^F signals, explore multimodal imaging fusion strategies, and conduct long-term toxicological studies in large animal models. Additionally, the aforementioned ESC-AGO-2 nanoparticles, among others, have T_1_-weighted signals due to ESIO, enabling real-time *in vivo* imaging and efficacy monitoring, showing strong potential for personalized therapy (Du et al., 2025).

Ultrasound-targeted microbubble destruction (UTMD) is an innovative platform technology that integrates ultrasound imaging with targeted therapy. Its core is the use of gas-filled microbubbles as carriers. When exposed to low-frequency ultrasound (LFUS), microbubbles oscillate stably (stable cavitation), facilitating nanoparticle absorption efficiency and tissue/organ specificity (Zhao et al., 2016a). Gao et al. prepared perfluoropropane (C_3_F_8_)-filled, polyethyleneimine-doped PLGA nanobubbles (CPPNBs) using a double emulsion evaporation method, and electrostatically adsorbed FGF21 (Gao et al., 2021). The gas inside the nanobubbles makes them excellent ultrasound contrast agents for non-invasive real-time monitoring of nanoparticle distribution in the heart. Under LFUS irradiation, the nanobubbles undergo cavitation, transiently increasing vascular and cell membrane permeability and triggering site-specific release of FGF21. The study showed that this strategy significantly enhanced FGF21 delivery efficiency to the diseased myocardium, synergistically downregulating key markers of cardiac hypertrophy, fibrosis, and apoptosis, achieving effective prophylactic treatment in a DCM mouse model. Limitations of this system include that FGF21 release is highly dependent on ultrasound parameters, with individual differences making parameter optimization complex, and the long-term (>8 weeks) biosafety and potential cumulative effects of repeated ultrasound irradiation on the myocardium remain unclear. Future developments could include closed-loop adaptive ultrasound systems that automatically adjust irradiation parameters based on real-time contrast feedback, or design ultrasound/ROS dual-responsive nanobubbles to reduce dependence on external equipment, and advance compatibility evaluation with clinical-grade ultrasound equipment.

Overall, current studies have validated the great potential of theranostics in DCM from different perspectives, ranging from detection platforms providing dynamic metabolic information to carriers combining imaging and targeted therapy. However, related research is still limited, with relatively few *in vivo* experiments or comparisons with classical diagnostic methods. Furthermore, the field still faces challenges such as high complexity of clinical translation, relatively single information dimensions, and the absence of an intelligent feedback loop. Future efforts should focus on multimodal integration of imaging and sensing technologies, establishment of standardized evaluation systems, and development of closed-loop therapeutic strategies based on biomarker feedback, thereby promoting a shift in DCM management from passive treatment to active intervention and early prevention.

## Application of nanoparticles by material type in DCM

In the previous section, we systematically reviewed the core advantages of nanoparticles in improving pharmacokinetics, enhancing drug stability and bioavailability, achieving targeted delivery, responsive release, and integrating diagnosis and treatment from a functional perspective. However, there are significant differences in the physicochemical properties, *in vivo* behavior, safety, and applicable scenarios of nanoparticles composed of different materials, which directly affect their conversion prospects in DCM treatment. Therefore, this section focuses on material types and discusses the research progress of polymer nanoparticles, liposomes, inorganic nanoparticles, and extracellular vesicles in DCM. The mechanism of action of antioxidant, anti-inflammatory and other drugs is not the focus of our discussion. Among them, antioxidant, anti-inflammatory, and anti fibrotic research is currently the most concentrated direction. In contrast, there are relatively limited reports on nano intervention strategies for cardiac energy metabolism reprogramming. Therefore, this section does not elaborate on metabolic regulation nano strategies, only briefly mentioning examples involving metabolic regulation in representative studies of various material types, and listing them as one of the important directions for future research. Table 1 summarizes and compares the key parameters of representative studies for each type.

**TABLE 1:** Main types of nanoparticles applied in DCM and their characteristics.

Nanoparticle type	Administration route	Encapsulated drug	Size	Cardiac function improvement	Toxicity	Source
Polymeric (chitosan-coated)	Oral	Ginsenoside Rb3	∼290 nm	LVEF increased by 37%	MTT assay showed no significant inhibition of fibroblast viability	Zhang et al. (2021a)
Liposomes (PEGylated ginkgo flavone glycosides)	Intraperitoneal injection	Ginkgo flavone glycosides	∼142.0 nm	LVEF increased by 81.3%	CCK-8 assay showed no significant cytotoxicity	Gao et al. (2025)
Inorganic Nanoparticles (metallic: gold)	Intravenous injection (tail vein)	miR155 antagonist (antago-miR155)	10 nm (bare); 20–40 nm (after conjugation)	LVEF recovered from ∼42% to ∼62%	Systemic toxicity has not been systematically evaluated	Jia et al. (2017)
Inorganic Nanoparticles (non-metallic: titanium oxide)	Intravenous	None (catalytic antioxidant)	<100 nm (thickness ∼5 nm)	LVEF recovered from 53% to near normal	CCK-8 assay showed no significant cytotoxicity	Yao et al. (2025)
Extracellular vesicles (ADSC-EVs)	Intravenous injection (tail vein)	Endogenous miRNA/protein	30–1000 nm	LVEF increased by approximately 20%	CCK-8 assay showed no significant cytotoxicity	Zhang et al. (2025)

LVEF: left ventricular ejection fraction; MTT: 3-(4,5-dimethylthiazol-2-yl)-2,5-diphenyltetrazolium bromide; PEG: polyethylene glycol; CCK8: Cell Counting Kit-8; ADSC-EVs: Extracellular vesicles derived from adipose-derived stem cell.

### Polymeric nanoparticles

Polymeric nanoparticles, owing to their good biodegradability, biocompatibility, and surface modification flexibility, have emerged as a representative platform for drug delivery in DCM (Vairaperumal et al., 2025). Commonly used materials include PLGA and chitosan, which can improve drug efficacy through passive or active targeting. Given the complex pathological mechanisms of DCM, several studies have used polymeric nanoparticles to deliver different therapeutic agents. Their common feature is the utilization of sustained release, targeting, and multi-mechanism synergy of nanocarriers, but they differ significantly in drug type, target, and therapeutic strategy.

Polymeric nanoparticles can be synthetically modified to deliver various drugs, including small molecules and peptides. Li et al. constructed dapagliflozin (DAPA)-loaded PLGA-thioketal (TK)-PEG nanoparticles designed for precise therapy through ROS-responsive release (Li et al., 2025). The nanoparticles exhibited a mean diameter of 66.1 nm and demonstrated an encapsulation efficiency of 86.11%. Under pH 6.5 or H_2_O_2_ conditions, the cumulative release of DAPA@PLGA-TK-PEG at 48 h (83.21%) was significantly higher than that of unmodified particles (73.56%). Compared with free DAPA, this nanoformulation more effectively attenuated myocardial fibrosis and oxidative stress markers (malondialdehyde and protein carbonyl, *p* <0.001) in DCM mice and high-glucose-induced cardiomyocyte models, while also elevating superoxide dismutase and catalase levels (*p* <0.001). In a different study, Zhang et al. focused on nanoformulation of peptide drugs, using PLGA to encapsulate a glucagon-like peptide-1 (GLP-1) nanocomposite peptide (size 40–60 nm) (Zhang et al., 2021b). In a rat cardiac injury model, the nanocomposite peptide produced dose-dependent reductions in serum LDH and cardiac troponin I (cTnI) levels (*p* <0.01), lowered malondialdehyde content, and attenuated cardiomyocyte programmed death through elevation of the Bcl-2/Bax ratio along with enhancement of mitochondrial DNA repair. Both of the above studies used PLGA as a carrier, but the former used a ROS-responsive linker to achieve drug release triggered by the lesion microenvironment, whereas the latter focused on sustained-release protection of the peptide.

Besides synthetic polymers, naturally derived chitosan also exhibits unique drug delivery potential, particularly for oral administration. Zhang et al. constructed ginsenoside Rb3-loaded nanoparticles (NpRb3) using chitosan-sodium tripolyphosphate, which exhibited an average diameter of approximately 290 nm (Zhang et al., 2021a). In a rat myocardial fibrosis model, 4 weeks of oral NpRb3 treatment significantly reduced serum cardiac troponin T (cTnT) and creatine kinase-MB (CK-MB) levels (*p*<0.001), increased LVEF (37%) and LVFS (49%), and reduced collagen deposition. Mechanistically, NpRb3 improved myocardial energy metabolism and mitochondrial function by targeting the PPARα pathway, thereby contributing to the inhibition of myocardial fibrosis. This study demonstrated the dual advantages of chitosan nanoparticles in improving oral bioavailability and promoting cellular uptake through positive charge, while also highlighting their potential for metabolic regulation in DCM.

Moreover, beyond functioning as drug delivery platforms, polymeric nanoparticles can generate synergistic therapeutic effects through their inherent constituents. Mohamed et al. prepared chitosan-stabilized selenium nanoparticles (CTS-Se-NPs) with a size of 6–22 nm (Mohamed et al., 2021). Using a type 2 diabetic rat model, 8 weeks of CTS-Se-NP treatment combined with metformin significantly lowered fasting glycemia and circulating insulin levels, while also mitigating cardiac histopathological damage. They reduced pro-inflammatory factors and malondialdehyde, increased antioxidant enzyme activities, and regulated apoptosis-related genes (downregulated caspase-3 and Bax, upregulated Bcl-2). Compared with the strategy of simply serving as a carrier, chitosan in this study not only stabilized the selenium nanoparticles but also enhanced mucosal adhesion and cellular uptake through its positive charge, reflecting a synergistic effect between the carrier material and the active component.

In summary, polymeric nanoparticles have significant advantages in DCM treatment: good biocompatibility, ability to achieve sustained controlled release and pathological microenvironment-responsive release. Nevertheless, current investigations remain largely at the preclinical stage, facing challenges such as inter-batch reproducibility during industrial scale-up, modest active targeting efficiency, and insufficient long-term safety data. Future research should summarize common mechanisms and develop differentiated nanomedicines targeting different pathological aspects.

### Liposomes

Liposomes are spherical vesicles whose phospholipid bilayer architecture closely mimics that of biological membranes. They exhibit excellent biocompatibility, low immunogenicity, and favorable biodegradability, with significantly lower toxicity than most polymeric and inorganic nanocarriers (Umar et al., 2025). In terms of drug loading capacity, liposomes can simultaneously encapsulate water-soluble and fat-soluble drugs owing to their hydrophilic core and hydrophobic bilayer, a dual-loading property that is difficult to achieve with polymeric nanoparticles (which mainly rely on hydrophobic interactions) or inorganic nanoparticles (Profire et al., 2025). Moreover, liposome surfaces can be easily PEGylated to prolong circulation half-life, and can also be conjugated with targeting ligands for active targeting, with relatively mature preparation processes (Eeda et al., 2025). Since the approval of the first PEGylated liposome, Doxil®, in 1995, liposomes have accumulated rich clinical translation experience in oncology and vaccines. In DCM treatment, several studies have utilized the above properties of liposomes to deliver different therapeutic agents to improve cardiac function by optimizing particle size, surface modification, and administration route.

Particle size is a key parameter affecting the passive targeting efficiency of liposomes. Gao et al. formulated PEGylated liposomes encapsulating ginkgo flavone glycosides (GFGs), which had a mean diameter of 142.0 nm and an encapsulation efficiency of 82% (Gao et al., 2025). This size favors passive targeting through the EPR effect in the damaged myocardium. In a DCM mouse model, this formulation combined with UTMD significantly increased LVEF and reduced myocardial fibrosis and apoptosis. The mechanism involved upregulation of silent information regulator 1 (SIRT1), inhibition of TSPAN4 transcription, and improvement of energy metabolism. Zhang et al. used a thin-film dispersion method to prepare PEGylated liposomes loaded with coenzyme Q10, with a mean diameter of 103.2 nm and an encapsulation efficiency as high as 87.45%, ensuring effective drug loading (Zhang et al., 2024). After 12 weeks of combined UTMD intervention, LVEF recovered from 53.40% in the model group to 80.76%. The mechanism involved antioxidant effects (increased SOD, decreased MDA) and anti-apoptotic effects (regulation of Bcl-2/Bax/caspase-3).

Surface modification strategies can further endow liposomes with active targeting or long-circulation capabilities. Ji et al. prepared astragalus polysaccharide liposomes with a particle size of only 14.29 nm. Although not PEGylated, the ultra-small size enabled tissue penetration, significantly reduced blood lipids, upregulated FAT/CD36 expression, and improved fatty acid metabolism (Ji et al., 2023). Zhao et al. loaded basic fibroblast growth factor into PEGylated liposomes, using PEGylation to prolong circulation half-life, and combined with UTMD for early intervention in DCM rats for 12 weeks. The results showed significantly improved cardiac function and increased microvessel density, which operates through stimulation of the PI3K/AKT signaling axis, thereby suppressing programmed cell death and facilitating blood vessel formation (Zhao et al., 2016b).

The choice of administration route also affects clinical translation potential. All of the above studies employed injection-based administration, whereas Alanazi et al. evaluated a commercially available oral liposomal formulation of resveratrol (size 159.33 nm, encapsulation efficiency 65.83%). After 5 weeks of oral administration, compared with an equal dose of free resveratrol, the liposomal formulation further lowered serum LDH and calcium levels and attenuated myocardial pathological injury. These effects were attributable to suppression of NF-κB activation and an anti-apoptotic mechanism (Alanazi et al., 2024).

In summary, liposomes, with their tunable size, high encapsulation efficiency, PEGylation modification, and various administration routes, provide a flexible and efficient delivery platform for poorly soluble small molecules, polysaccharides, and proteins in DCM treatment. However, current limitations include complex preparation processes, need for improved batch consistency, drug leakage in some formulations, and insufficient long-term toxicity data.

### Inorganic nanoparticles

Unlike polymeric nanoparticles that rely on degradation-controlled release or liposomes limited by phospholipid stability, inorganic nanoparticles, with their inorganic crystal skeleton, exhibit excellent stability in biological fluids and are not prone to burst release (Luo et al., 2026). Their high specific surface area and quantum size effects facilitate integration of therapeutic and diagnostic modules, and their surface chemistry is highly tunable, thereby overcoming the limitations associated with the complex functionalization of polymeric particles and the aggregation propensity of surface-modified liposomes. Metal-based (gold, silver, iron oxides) nanoparticles mainly utilize surface plasmon resonance, magnetic properties, and ease of modification; non-metal-based (selenium, silicon dioxide, molybdenum disulfide, carbon nitride) nanoparticles rely more on catalytic activity or semiconducting properties (Jia et al., 2017; Wei et al., 2021; Wang et al., 2023; Galagudza et al., 2010). In DCM, these properties provide unique advantages for theranostics.

#### Metal nanoparticles

Metal nanoparticles can either exert intrinsic anti-inflammatory and antioxidant effects or be surface-modified for targeting. Aljabali et al. utilized the anti-inflammatory properties of AuNPs, preparing 50 nm AuNPs (Aljabali et al., 2021). In streptozotocin (STZ)-induced DCM rats, intraperitoneal injection of AuNPs significantly reduced blood glucose and left ventricular collagen deposition, downregulating TGF-β1, TNF-α, and VEGF-A. Complementarily, Atale et al. green-synthesized silver nanoparticles (AgNPs, 40–100 nm, zeta potential −19.6 mV), whose surface plant polyphenols conferred strong antioxidant activity (Atale et al., 2017). The study showed that AgNPs treatment for 48 h reversed cell hypertrophy and restored nuclear morphology, with DPPH and ABTS inhibition rates reaching 66% and 86%, respectively.

Beyond the intrinsic activity of metals, iron oxide nanoparticles also combine superparamagnetism and ligand-conjugating capabilities, making them suitable for MRI-guided targeted therapy. Du et al. constructed ESIO modified with CHP and the mitochondria-targeting peptide SS-31, loaded with AGO-2 protein *db/db* mice, this probe targeted myocardial mitochondria, and T_1_-weighted MRI showed significant signal enhancement in the myocardium within 30 min. After treatment, ROS was reduced, mitochondrial structure was restored, and PGC-1α was upregulated. Similarly, Liu et al. conjugated SPIO with CHP and MMP2 antibody, and taking advantage of upregulated MMP2 in the myocardium of 12-week *db/db* mice, T_2_-weighted MRI showed negative enhancement signals (T2% >20%) 30 min after probe injection, earlier than clinical T_1_ mapping, achieving ultra-early diagnosis of fibrosis (Liu et al., 2024).

#### Non-metal nanoparticles

Non-metal inorganic nanoparticles focus more on catalytic or sensing functions. Unlike metals, titanium-based materials can continuously scavenge ROS through redox cycling within the crystal lattice. Yao et al. synthesized two-dimensional TiO_x_ (OH)_4–2x_ nanosheets (<100 nm) that catalyze H_2_O_2_ disproportionation via Ti^IV^/Ti^III^ redox pairs (Yao et al., 2025). In high-glucose H9c2 cells, 50 ppm treatment for 24 h reduced ROS, restored mitochondrial membrane potential, and increased ATP, GSH, and decreased MDA. In DCM mice, the nanosheets recovered LVEF from 53% to near normal and induced M1-to-M2 macrophage polarization. Selenium nanoparticles (SeNPs) primarily exert antioxidant effects by mimicking selenoenzymes. Lotfy et al. treated STZ-diabetic rats with 80 nm SeNPs (0.1 mg/kg/d, oral gavage for 4 weeks), significantly reducing serum CK-MB, LDH, cTnI, cTnT, and CRP, with mechanisms involving downregulation of miR-21 and miR-328 and inhibition of the TGF-β1/Smad and JAK/STAT pathways (Lotfy et al., 2023).

In the diagnostic field, the semiconducting or optical properties of non-metal nanomaterials are used to construct highly sensitive sensors. Abraham et al. used MoS_2_ nanosheets and a terbium-citrate complex to construct a FRET probe for “turn-on” detection of brain natriuretic peptide (BNP), achieving a detection limit of 3.87 pg/mL (Abraham et al., 2024). Kumar et al. used carboxylated graphitic carbon nitride (g-C_3_N_4_) nanoparticles for electrode modification, coupled with ECL, to enable simultaneous detection of H_2_O_2_, glucose, and lactate released from H9c2 cells. The obtained detection limits were 0.91 μM for H_2_O_2_, 642 μM for glucose, and 135 μM for lactate (Kumar et al., 2025).

In summary, inorganic nanoparticles have both therapeutic and diagnostic functions in DCM. Metal-based nanoparticles, with their ease of modification, magnetic properties, and intrinsic activity, achieve targeted drug delivery, anti-inflammatory and anti-fibrotic effects, and MRI early diagnosis. Non-metal-based nanoparticles directly scavenge ROS, regulate inflammation, or construct highly sensitive sensing platforms using catalytic cycling, enzyme-mimicking, or photoelectric properties. Notably, the safety profiles of inorganic nanoparticles warrant careful consideration when translating these promising therapeutic strategies into clinical practice. Although Aljabali et al. demonstrated cardioprotective effects of 50 nm AuNPs in STZ-induced diabetic rats, the study did not perform *in vitro* cytotoxicity assays (e.g., CCK-8 or MTT) nor systematically evaluate *in vivo* organ toxicity, and thus the safety margin between the therapeutically effective dose and the maximum tolerated dose remains undefined (Aljabali et al., 2021). In contrast, zinc oxide nanoparticles have provided dose-dependent evidence: a 3 mg/kg dose exerted cardioprotective effects in diabetic rats, whereas a 10 mg/kg dose aggravated cardiac injury, suggesting a relatively narrow therapeutic window (Asri-Rezaei et al., 2017). For TiO_x_ (OH)_4–2x_, although CCK-8 assays confirmed no significant cytotoxicity within the 25–50 ppm concentration range, *in vivo* safety evaluation was primarily focused on the heart without systematic assessment of other organs (Yao et al., 2025). Collectively, current safety data for inorganic nanoparticles in DCM therapy remain insufficient, and the safety window between therapeutically effective doses and maximum tolerated doses has not been clearly established. Future studies should systematically incorporate *in vitro* cytotoxicity screening (CCK-8/MTT assays) and *in vivo* multi-organ toxicity evaluation (serum biochemical parameters, organ coefficients, histopathology) into nanocarrier design to define the safety window and facilitate clinical translation.

### Extracellular vesicles (EVs)

Extracellular vesicles (EVs) are cell-secreted, membrane-enclosed structures with diameters of 30–200 nm. These vesicles transport bioactive molecules, including proteins, mRNA, and miRNA, thereby functioning as mediators of intercellular communication (Shyu et al., 2025). In contrast to synthetic nanoparticles, extracellular vesicles offer inherent benefits, including a natural origin, minimal immunogenicity, and the capacity to cross biological barriers (Yin et al., 2026). In DCM treatment, EVs exert protective effects by regulating inflammation, fibrosis, and pyroptosis (Gan et al., 2025).

Zhang et al. isolated EVs from ADSCs. Transmission electron microscopy showed typical vesicular structures (30–1,000 nm) expressing CD9, CD81, and Alix (Zhang et al., 2025). In STZ-induced DCM rats, tail vein injection of Adipose-derived stem cell- Extracellular vesicles (ADSC-EVs) (200 μg, twice weekly for 4 weeks) significantly reduced fasting blood glucose, LVIDs, and LVIDd, and increased LVEF and LVFS. Masson staining revealed alleviated myocardial fibrosis, and Western blot confirmed that ADSC-EVs inhibited pyroptosis and reduced the expression of alpha-smooth muscle actin and collagen type I. mRNA sequencing and knockdown experiments revealed that ADSC-EVs suppressed the NLRP3/Caspase-1 pyroptosis pathway by downregulating chitotriosidase 1.

Unlike mammalian EVs, plant-derived exosome-like nanoparticles (ELNs) offer the benefits of high production yields and rapid isolation procedures. Peng et al. isolated and purified Salvia miltiorrhiza-derived ELNs (SM-ELNs), with a mean diameter of 103.24 nm and a surface charge of −24.56 mV (Peng et al., 2025). In a mouse model of DCM established by a high-fat diet in combination with STZ administration, tail vein injection of SM-ELNs (10 mg/kg for 14 days) significantly led to enhanced cardiac function, as reflected by elevated EF and FS, along with attenuation of myocardial fibrosis and cardiac hypertrophy. Mechanistically, SM-ELNs upregulated the E3 ubiquitin ligase NEDD4 in macrophages, which facilitated ubiquitin-mediated breakdown of serum/glucocorticoid-regulated kinase 1 (SGK1), thus suppressing pyroptosis in macrophages mediated by the NLRP3 inflammasome. *In vitro* experiments substantiated that SM-ELNs reversed the increases in NLRP3, IL-1β, IL-18, and GSDMD induced by lipopolysaccharide (LPS)/ATP in RAW264.7 cells, and NEDD4 knockdown or SGK1 overexpression abolished their inhibitory effects.

Lin et al. focused on the regulation of the TGF-β1/Smad2 pathway by mesenchymal stem cell (MSC)-derived EVs (Lin et al., 2019). EVs were isolated from rat bone marrow MSCs (CD63 positive). In STZ-diabetic rats, tail vein injection of MSC-EVs (100 μg once weekly for 12 weeks) significantly reduced left ventricular collagen content and increased myocardial fatty acid transporter and fatty acid β-oxidation enzyme levels. qRT-PCR and Western blot showed that MSC-EVs downregulated the mRNA and protein expression of TGF-β1 and Smad2, indicating that they improve myocardial fibrosis and energy metabolism disorders through suppression of the TGF-β1/Smad2 signaling cascade.

In summary, as natural nano-delivery systems, extracellular vesicles have advantages such as low immunogenicity and multi-component synergy in DCM treatment. However, several obstacles hinder the clinical translation of EVs: the standardization of large-scale production, inter-batch reproducibility, purification yield, and long-term storage stability remain insufficient; drug encapsulation efficiency and surface targeting strategies for EVs require further refinement; additionally, the *in vivo* pharmacokinetic behavior and chronic safety profiles of EVs derived from different sources necessitate systematic evaluation. Future research should focus on engineering EVs to enhance targeting and therapeutic efficacy and promote their clinical translation.

## Challenges and future perspectives

Nanotechnology holds multifaceted promise for the diagnosis and therapy of DCM. Through rational design, nanoparticles can effectively improve drug circulation characteristics, achieve myocardial targeted delivery, respond to the pathological microenvironment for on-demand release, and integrate diagnostic and therapeutic functions. Polymeric nanoparticles, owing to their good biodegradability and surface modification flexibility, are the most widely used platform. Liposomes, with their structure similar to cell membranes, have excellent biocompatibility and amphiphilic drug-loading capacity. Inorganic nanoparticles, with their stable crystal skeleton and unique catalytic/optical properties, have unique advantages in antioxidant therapy and molecular imaging. Extracellular vesicles, as natural delivery systems, are attracting attention for their low immunogenicity and multi-component synergy. These platforms have demonstrated efficacy in antifibrotic therapy, anti-oxidation, anti-apoptosis, and metabolic regulation in various DCM animal models.

Research on nanoparticles in cardiovascular diseases did not begin with DCM. In other cardiac diseases, including myocardial infarction, atherosclerosis, and heart failure, nanotechnology has accumulated richer experience. For example, in post-myocardial infarction repair, nanoparticles carrying anti-inflammatory or pro-angiogenic factors can target the infarct border zone, reducing scar area and promoting neovascularization; some nanosystems have achieved myocardial regeneration by delivering stem cells or exosome mimetics (Yang et al., 2025a; Yang et al., 2025b; Wang et al., 2025a). In atherosclerosis, nanoprobes targeting plaque macrophages have been successfully used for early assessment of unstable plaques (Song et al., 2026). In contrast, DCM has unique pathological features: the lesions are diffuse rather than focal, the disease course is protracted, and it originates from metabolic disorders rather than acute ischemia (Liu et al., 2025). Therefore, DCM imposes higher requirements on nanoparticle targeting: not only must they recognize myocardial tissue, but they must also distinguish between healthy and diseased areas, and even require subcellular (e.g., mitochondrial)-level precision deliver (Du et al., 2026). Moreover, the chronic nature of DCM demands that nanosystems possess long-term stability and safety for repeat administration, which imposes stricter standards on material biodegradability and immunogenicity.

Notably, the vast majority of current nanoparticle-based therapeutic studies in DCM have failed to incorporate sex as a biological variable in their experimental designs. Epidemiological evidence indicates that although premenopausal women have a lower risk of cardiovascular disease than age-matched men, this female advantage is abolished by diabetes, diabetic women exhibit a 5.1-fold increased risk of heart failure compared with non-diabetic women, whereas diabetic men show only a 2.4-fold increase (Kannel et al., 1974). Animal studies have similarly demonstrated that female diabetic mice develop more severe myocardial pathology, characterized by both systolic and diastolic dysfunction as well as pronounced cardiac metabolic perturbations (Karatzia et al., 2025). Although estrogen exerts cardioprotective effects through pathways such as Akt-FoxO1 signaling, its protective role is attenuated or even reversed under diabetic conditions (Yan et al., 2022; Leffler and Abdel-Rahman, 2019). From the perspective of nanomedicine delivery, sex differences can influence protein corona formation, biodistribution, pharmacokinetics, immune responses, and cellular uptake of nanoparticles (Hajipour et al., 2021; Sharifi et al., 2021). However, current DCM nanomedicine research predominantly employs only male animal models, overlooking the potential sex-dependent differences in nanoparticle clearance rates and myocardial uptake. Future studies should include both male and female animals to systematically analyze the influence of sex on nanoparticle pharmacokinetics, myocardial targeting efficiency, and therapeutic responses, and to explore the possibility of tailoring nanocarrier design for sex-specific optimization.

Furthermore, the animal models predominantly employed in current DCM nanomedicine research present notable limitations that warrant critical consideration. The vast majority of studies rely on STZ induced type 1 diabetes models, yet clinical DCM predominantly affects patients with type 2 diabetes mellitus (T2DM), a condition characterized by insulin resistance, obesity, and hyperlipidemia (DeFronzo, 2010). Emerging evidence demonstrates that STZ-induced T1DM and T2DM models exhibit fundamentally different patterns of cardiac remodeling, functional alterations, and whole-transcriptome profiles—T1DM is characterized by more pronounced cardiomyocyte apoptosis, reactive hypertrophy, and fibrosis, accompanied by elevated cardiac oxidative stress and DNA damage (Marino et al., 2023). Moreover, clinical DCM typically develops over decades of chronic metabolic dysregulation, whereas most animal models only recapitulate short-term hyperglycemia over weeks to months, failing to capture the time-dependent and multifactorial nature of the human disease (Lezoualc’h et al., 2023). Consequently, direct extrapolation of findings from STZ-based nanoparticle studies to clinical T2DM-DCM must be approached with caution. Future investigations should prioritize the use of animal models that more faithfully recapitulate the pathophysiological features of human T2DM-DCM, including genetically modified obese diabetic models (e.g., *db/db* mice, ZDF rats), diet-induced obesity combined with low-dose STZ (HFD + STZ), and multifactorial comorbid models incorporating additional cardiovascular risk factors such as aging, hypertension, and hyperlipidemia. Furthermore, cross-validation of therapeutic efficacy and safety across at least two distinct animal models is strongly recommended to enhance the robustness and translational relevance of research findings.

Therefore, drawing on successful experiences of nanoparticles in other cardiovascular diseases (e.g., myocardial infarction, atherosclerosis) and considering the diffuse lesions, chronic course, and metabolism-dominated pathology of DCM, future research may achieve breakthroughs in the following directions.

In terms of material design, biomimetic nanoparticles are expected to be a key strategy to address rapid clearance and low targeting efficiency (Gu et al., 2025). Biomimetic nanoparticles coated with membranes derived from red blood cells, macrophages, or stem cells can effectively disguise themselves as endogenous entities, thereby markedly extending their circulation half-life and decreasing immunogenicity. Given the chronic inflammatory microenvironment of DCM, macrophage membrane-biomimetic particles could actively chemotax to the damaged myocardium, while stem cell membrane-biomimetic particles might mimic paracrine effects to synergistically deliver anti-inflammatory or pro-repair factors (Chen et al., 2022). In the future, combining with gene editing technology, specific chemokine receptors could be displayed on the membrane surface to further enhance lesion homing ability.

Targeting strategies need to be upgraded from single-ligand recognition to multi-dimensional synergy (Xu et al., 2025a). Biomarkers in DCM change dynamically during the disease course (e.g., upregulation of oxidative stress markers in the early stage, dominance of fibrosis markers in the late stage), and a single target may become ineffective due to feedback regulation. Designing multi-ligand nanoparticles that simultaneously recognize myocardial injury, inflammation, and oxidative stress may improve targeting accuracy (Ye et al., 2025). In addition, physical targeting methods such as magnetic guidance or ultrasound mediation can be combined with ligand modification to achieve spatiotemporally controllable directional enrichment.

Diversification of administration routes also deserves attention. Most current studies use intravenous or intraperitoneal injection, which, while achieving systemic distribution, still carry risks of non-specific accumulation. Oral administration is the most convenient but is only suitable for carriers with high stability, such as chitosan. Subepicardial or intramyocardial injection can be used for intraoperative local treatment (Wang et al., 2025b). Given the need for long-term medication in DCM, developing non-invasive modes such as long-acting sustained-release microspheres or transdermal patches may improve patient compliance.

The maturation of theranostic systems is another important direction. Currently, most related research in DCM focuses on *in vitro* sensing or single MRI imaging, lacking intelligent platforms that integrate multimodal imaging and synergistic therapy. In the future, near-infrared fluorescence, photoacoustic imaging, PET/CT, and other imaging modalities could be integrated, while carrying antioxidants, anti-fibrotic drugs, and gene therapy molecules to achieve “see and treat” (Nankivell et al., 2025; Lin et al., 2025). Combining with wearable devices and closed-loop feedback systems to dynamically monitor circulating biomarkers and trigger drug release will be the ultimate goal of intelligent diagnosis and treatment.

Safety evaluation and clinical translation are the core bottlenecks limiting the realization of nanotechnology. Existing studies are mostly small animal experiments, with severely insufficient data on the long-term toxicity, biodistribution, and metabolic clearance of nanoparticles. Future studies should use large animal models (e.g., pigs, dogs) for repeat dosing and long-term follow-up, systematically assessing nanoparticle accumulation and tissue damage in the heart, liver, kidney, spleen, and other organs. At the same time, unified quality control standards (e.g., size distribution, encapsulation efficiency, *in vitro* release behavior) and preclinical safety evaluation guidelines need to be established to facilitate the clinical translation of nanomedicines.

Furthermore, nanoparticles are not an isolated drug delivery technology; their combined application with local delivery systems such as hydrogels and myocardial patches will produce synergistic effects (Yang et al., 2025a). Hydrogels can be injected *in situ* onto the epicardium or into the myocardium, forming drug depots and filling fibrotic areas, with sustained release periods reaching weeks to months. Myocardial patches can provide physical support while loading nanoparticles for regional therapy (Xu et al., 2025c; Cristallini et al., 2025). In DCM, although lesions are diffuse, local intensive treatment of high-stress areas of the left ventricle (e.g., free wall, interventricular septum) still has clinical value. In the future, nanoparticles could be combined with hydrogels or patches to form macro-micro two-stage delivery systems: patches provide mechanical support and long-term sustained release, while nanoparticles achieve targeted transmembrane delivery; ultrasound- or magnetic-responsive nanoparticles could also be triggered by external fields for local release, forming a synergistic and controllable network with the patch.

In summary, nanotechnology has opened a new path for early diagnosis and precise treatment of DCM. Drawing on successful experiences in other cardiac diseases and addressing the unique pathological characteristics of DCM through biomimetic design, targeting upgrades, administration optimization, and safety validation, while exploring combined strategies with local systems such as hydrogels and myocardial patches, will be core directions for future research. Only through multidisciplinary integration, continuous material innovation, and rigorous translational research can the leap of nanoparticles from the laboratory to the bedside be ultimately achieved, benefiting the vast number of DCM patients.

## Conclusion

Nanoparticles, with their unique size effects, high specific surface area, surface modifiability, and multifunctional integration capabilities, offer a novel technological route for the early detection and precision therapy of DCM. Current studies have shown that different types of nanoplatforms—including polymeric nanoparticles, liposomes, inorganic nanoparticles, and extracellular vesicles—can improve the pharmacokinetic profiles of drugs, enhance targeted myocardial delivery, achieve on-demand release in response to the pathological microenvironment such as high glucose levels, oxidative stress, inflammation, and fibrosis, and exert beneficial effects on antioxidant, anti-inflammatory, antifibrotic, anti-apoptotic, and energy metabolism-regulating processes. At the same time, some functionalized nanosystems possess the potential to integrate molecular imaging, pathological monitoring, and drug delivery, thereby supporting the development of theranostic strategies for DCM. However, compared with other cardiovascular diseases such as myocardial infarction and atherosclerosis, DCM is characterized by diffuse lesions, a protracted course, metabolic dysregulation-dominated pathogenesis, and chronic progression, placing higher demands on nanoparticle targeting accuracy, long-term stability, repeat-dose safety, and controllable *in vivo* metabolism and clearance. Therefore, future research should, drawing on advances in nanotherapy for other cardiovascular diseases, further develop biomimetic, multi-ligand synergistic targeting, subcellular precision delivery, microenvironment-responsive release, and multimodal theranostic nanosystems, while also exploring their combined use with local or long-acting delivery platforms such as hydrogels, myocardial patches, and transdermal patches to improve treatment sustainability and patient compliance. At the same time, there is an urgent need to strengthen validation in large animal models, long-term toxicity evaluation, *in vivo* distribution and metabolism studies, batch-to-batch stability control, and standardized quality evaluation systems. In conclusion, nanoparticles show promising application prospects in the diagnosis and treatment of DCM, but their clinical translation still depends on continued breakthroughs in material design, targeting strategies, safety validation, and scalable preparation.
